# La luxation chronique du coude chez l'enfant: à propos de 20 cas

**DOI:** 10.11604/pamj.2014.18.348.5136

**Published:** 2014-08-29

**Authors:** Karima Atarraf, Mounir Arroud, Lamiae Chater, My Abderrahmane Afifi

**Affiliations:** 1 Service d'Orthopédie Pédiatrique, Faculté de Médecine et de Pharmacie, Université Sidi Mohammed Ben Abdullah, CHU Hassan II, Fès, Maroc

**Keywords:** Luxation négligée, coude, enfant, neglected dislocation, elbow, infant

## Abstract

La luxation négligée du coude chez l'enfant est peu fréquente dans les pays industrialisés, elle n'est pas en revanche rare dans notre contexte. Nous rapportons une série de 20 cas, colligée au service d'Orthopédie Pédiatrique du CHU HASSAN II de Fès, sur une période de 4 ans. En effet, 14 malades étaient de sexe masculin, avec un âge moyen de 8,5 ans. Le mécanisme principal était la chute d'olivier. Le délai de consultation était très variable. 19 enfants ont été traités traditionnellement. La raideur constituait le motif principal de consultation. Avec une flexion inférieure à 70°. Un seul malade avait présenté un syndrome de Volkmann. La luxation était isolée et postéro-externe dans la majorité des cas. La voie d'abord médiane postérieure était adoptée chez tous nos malades. 15 patients ont bénéficié d'une arthrolyse avec arthrodèse provisoire. La rééducation était systématique. Le résultat a été bon avec une flexion à 90° et un secteur de mobilité supérieur à 60° chez 9 de nos malades sur un recul de 3 ans. En définitive, la luxation invétérée du coude est une pathologie du milieu rural; la facilité d'accès au soin reste la meilleure prévention.

## Introduction

La luxation négligée du coude chez l'enfant est une atteinte peu fréquente dans les pays industrialisés, elle n'est pas en revanche rare dans notre contexte, elle représente l’évolution d'une luxation du coude non prise en charge dans les délais, elle est volontiers associée à une fracture.

## Méthodes

Nous rapportons une série de 20 cas de luxation négligée du coude chez l'enfant colligée au service d'Orthopédie Pédiatrique au CHU HASSAN II de Fès, sur une période de 4 ans, dont l'objectif est de discuter le traitement qui présente un des défis de la chirurgie.

## Résultats

**Figure 1:**
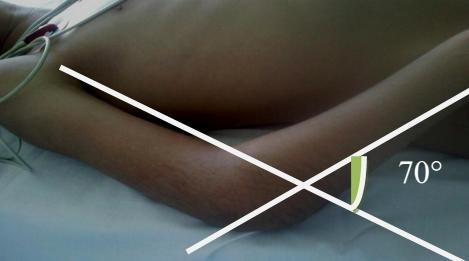
Secteur de mobilité fonctionnel chez un enfant de 6 ans à son admission bloqué à 70° par rapport au plan d'extension

**Figure 2:**
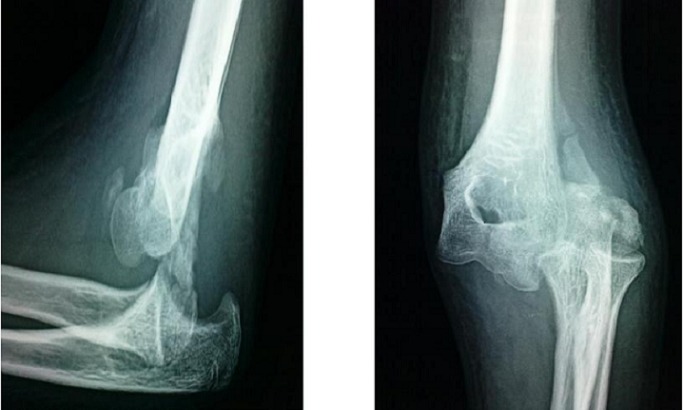
Luxation postéro latérale du coude négligée de 22 jours chez une fille de 14 ans, associée à une fracture du condyle externe et de l’épitrochlée

14 de nos malades étaient de sexe masculin, avec un âge moyen de 8,5 ans (6ans - 11 ans). 17 de nos malades provenaient du milieu rural (85%), avec comme mécanisme principal la chute d'un olivier. le côté droit était majoritairement atteint (60% des cas). Le délai de consultation était très variable allant de 15 jours à 2 ans, uniquement 8 de nos malades avaient consulté à un intervalle de 1 à 3 mois, et 9 de nos malades ont consulté après un délai de 3 à 6 mois. 19 enfants ont été traités traditionnellement par des attelles en roseau (JBIRA). La raideur constituait le motif principal de consultation associée à la déformation et à l'impotence fonctionnelle partielle du membre supérieur. Seulement deux de nos malades avaient un secteur de flexion situé entre 70°-90°, alors que la majorité représentée par 11 cas avait une flexion située entre 40°-70° (Figure 1). Un seul malade avait présenté un syndrome de Volkmann suite au traitement traditionnel et ayant consulté 2 semaines après le traumatisme. Aucun cas d'atteinte vasculo-nerveuse n'a été constaté. Sur les 20 cas étudiés, la luxation était isolée dans 13 cas, et associée à une ou plusieurs fractures dans 7 cas soit; dont 03 cas de fracture de l’épitrochlée, 02 cas de fracture du condyle latéral, dans un cas elle a été associée à la fois à une fracture de l’épitrochlée et du condyle externe (Figure 2), et dans un cas il s'agissait d'une fracture de l'olécrane. La luxation était postéro-externes dans 16 cas, et postérieure et postéro médiane dans 2 cas chacune. Tous nos patients avaient une raideur grave à très grave, et qui était le motif principal de consultation. La voie d'abord médiane postérieure était adoptée chez tous nos malades.

**Figure 3:**
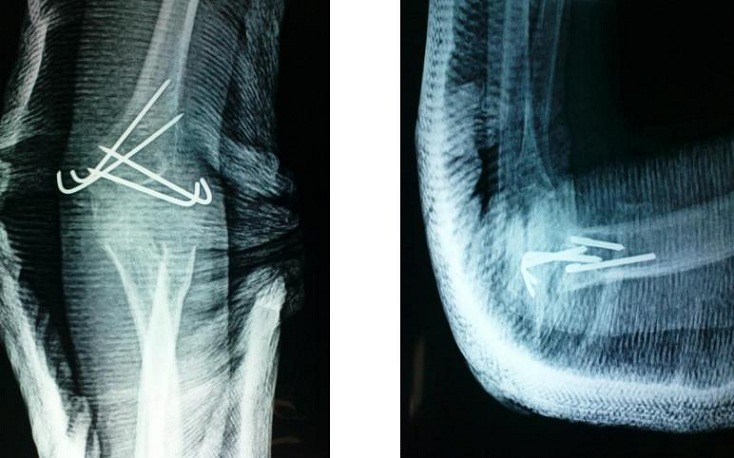
Clichés face et profil après la réduction chirurgicale

**Figure 4:**
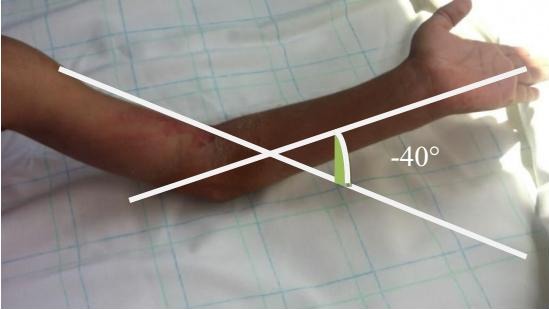
Résultat du secteur de mobilité en extension du coude à -40° après traitement chirurgical

**Figure 5:**
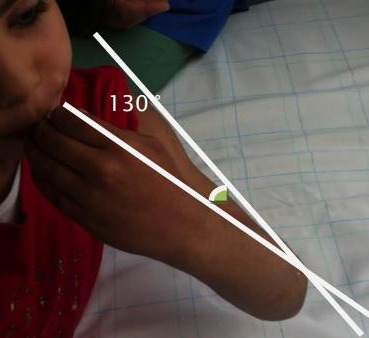
Résultat du secteur de mobilité en flexion du coude à 130°

Ainsi 5 cas ont bénéficié d'une arthrolyse simple, alors que 15 patients ont bénéficié d'une arthrolyse avec arthrodèse provisoire trans olécrano-huméral, avec une ostéosynthèse des fractures associées chaque fois qu'il y a une (Figure 3). 15 patients de notre série ont bénéficié d'une immobilisation plâtrée en postopératoire par Attelle plâtrée brachio-anté-brachio palmaire pendant une durée de 3 semaines. Alors que les 5 autres ont bénéficié d'attelles de postures par manque d'arthromoteur. La rééducation postopératoire était d'une importance majeure, elle se faisait en deux phases: Une phase précoce: débutée vers le 10^ème^ jour du postopératoire; reposant sur l'usage de deux attelles de posture, une en flexion maximale, et l'autre en extension maximale, les positions étant renouvelées chaque 6 heures. Ce protocole a été réalisé chez 5 de nos malades. Et une phase tardive débutée à la 3^ème^ semaine, chaque fois qu'il y a une fixation par broche. 9 de nos malades avait un résultat bon avec une flexion à 90° et un secteur de mobilité >à 60° (Figure 4, Figure 5), il a été moyen chez 9 autres, avec une flexion >90°et un secteur de mobilité < à 60°. Alors que dans 02 cas le résultat a été mauvais avec un coude bloqué à 90°. En fonction de la position initiale, les bons et moyens résultats ont été retrouvés chez le groupe de malades ayant une flexion comprise entre 40°et 70°, et chez les patients pris en charge avant 6 mois, et rééduqués précocement.

## Discussion

La luxation négligée du coude chez l'enfant est une pathologie de prise en charge difficile et laborieuse, il s'agit d'une pathologie des pays en voie de développement. Elle touche avec prédilection le garçon issu du milieu rural [1]. Le délai de consultation était très variable selon les séries, allant de 15 jours à 2 ans dans notre série, de 6 mois à 16 mois et de 2 mois à 18 mois dans les autres séries [1, 2]. L’énorme pourcentage de luxations chroniques du coude traitées traditionnellement par « JBIRA » est concordant avec les résultats des autres séries [1, 2], et ne peut être expliqué que par les croyances, l'analphabétisme des parents et le niveau socio économique bas retrouvé dans 60% des cas. la luxation était postéro-externe chez 16 patients, ce qui concorde avec les résultats de l’étude de M.M. AlZohairy [3]. Alors qu'elle était majoritairement postéro-médiane. Dans la série de Z.F.El Alami [1]. Les associations lésionnelles à la luxation du coude étaient de 35%, rejoignant la littérature ou sont situés aux alentours de 40%. Les complications les plus fréquentes dans la littérature sont les atteintes nerveuses en particulier l'atteinte du nerf cubital, qui présente 6% à 8% des complications; suivies des lésions cutanées à type d'ouverture cutanée ou de plaies infectées [1, 2]. Les complications vasculaires apparaissent exceptionnelles sauf pour Y. Teklali [2] qui rapporte un cas d'atteinte de l'artère humérale. Dans notre série aucune complication n'a été retrouvée sauf un cas de syndrome de Volkmann secondaire à un traitement traditionnel par « JBIRA ». Le secteur fonctionnel en flexion était satisfaisant dans 02 cas seulement avec une flexion située entre 70°-90°. 18 enfants avaient une flexion inferieure à 70°. Le déficit en extension était inferieur à 10° chez seulement 4 cas. Ce qui concorde avec les résultats de l’étude de Z.F.El Alami [1].

Les données de la littérature [1, 2, 4] rapportent quelques cas d'abstention thérapeutique vu le secteur de mobilité qui était jugé satisfaisant sur le plan fonctionnel, même avec un coude luxé. Dans notre série, toutes les luxations négligées du coude ont été opérées vu la raideur très importante, le délai de consultation dépassant 3 mois et la mobilité située hors secteur fonctionnel. L'objectif du traitement était de réduire l'articulation du coude surtout qu'il s'agit d'un coude en croissance, d'améliorer le secteur de mobilité et au moins de mettre le coude en position de fonction. Tous les auteurs sont unanimes en ce qui concerne la voie d'abord, c'est ainsi que la voie postérieure transtricipital avec désinsertion et allongement du triceps, donnant entière satisfaction, en permettant une dissection aisée du nerf cubital et une libération facile des attaches internes et externes [1–3, 5]. Dans notre série, l'embrochage était essentiellement trans-olécrano huméral chez 15 cas soit 75% ce qui converge vers les résultats de la littérature [1, 2]. Une ostéosynthèse complémentaire par des broches de kirshner était réalisé chaque fois qu'il y a une fracture associée. Généralement l'ablation des broches se fait vers la 3^ème^ semaine pour les broches transolécrano-huméral, et à la 6^ème^ pour les broches d'ostéosynthèse dans les fractures associée [6]. Thomas [7], Sharma [8] pensent que la période de fixation doit être déterminée en fonction de l’âge et le type de la fracture traitée et ils ont proposé un intervalle entre 3 et 5 semaines. Pour toutes les études de la littérature, l'immobilisation post-opératoire repose sur une attelle plâtrée postérieure brachioantébrachio palmaire pour une période variable allant de 10 jours à 21 jours, comme pour la majorité de nos patients, L'immobilisation se faisait également par des attelles de posture en flexion et en extension en cas de rééducation débutée précocement [9].

Dans notre série, la rééducation était débutée dans la majorité des cas vers la 3^ème^ semaine, ce qui concorde avec la littérature [1–3]. Chez 6 malades, la rééducation était débutée aussi précocement vers la 1^ère^ semaine à l'aide d'attelle de posture (au lieu de l'arthromoteur) permettant une mobilisation passive précoce. Dans notre série; un seul cas de récidive était enregistré, contrairement à la littérature [1–3] qui rapporte quelques cas de paralysie ulnaire et d'infection sur broche. dans notre série, plus le délai de consultation est inférieur à 3 mois, plus les résultats du traitement sont bons, ce qui concorde avec les résultats de la littérature [1, 2, 5]. Les meilleurs résultats étaient enregistrés avec les luxations isolées, ce qui a été approuvée par toutes les études menées à ce propos [1–3], et la rééducation parait plus rentable si a été débutée précocement. L'indication chirurgicale doit absolument être pesée en tenant compte du type de lésions associées, du délai de la consultation,de l’âge du patient, du secteur de mobilité lors de la première consultation et après de la rééducation [10].

## Conclusion

Les luxations du coude sont des traumatismes de bon pronostic, à condition d'une prise en charge précoce, adaptée et cohérente. Par conséquent tout retard de prise en charge va transformer une luxation fraiche en une luxation négligée dont le traitement sera difficile avec des résultats imprévisibles. Le pronostic étant marqué par la raideur qui dépend alors de plusieurs facteurs à savoir le délais de prise en charge et les lésions associées.
